# Co-Morbidities and Sex Differences in Long-Term Quality-of-Life Outcomes among Patients with and without Diabetes after Total Knee Replacement: Five-Year Data from Registry Study

**DOI:** 10.3390/jcm9010019

**Published:** 2019-12-19

**Authors:** Michelle Tew, Michelle M. Dowsey, Annabelle Choong, Peter F. Choong, Philip Clarke

**Affiliations:** 1 Centre for Health Policy, Melbourne School of Population and Global Health, The University of Melbourne, Carlton, VIC 3053, Australia; Philip.Clarke@ndph.ox.ac.uk; 2 Department of Orthopaedics, St. Vincent’s Hospital Melbourne, Fitzroy, VIC 3065, Australia; mmdowsey@unimelb.edu.au (M.M.D.); Annabelle.Choong@svha.org.au (A.C.); pchoong@unimelb.edu.au (P.F.C.); 3 Department of Surgery, The University of Melbourne, St. Vincent’s Hospital Melbourne, Fitzroy, VIC 3065, Australia; 4 Health Economics Research Centre, Nuffield Department of Population Health, The University of Oxford, Oxford OX3 7LF, UK

**Keywords:** quality-of-life, joint surgery, sex differences, patient-reported outcomes, co-morbidities

## Abstract

Improved understanding of quality-of-life (QoL) outcomes can provide valuable information on intervention effectiveness and guide better patient care. The aim of this study was to examine whether QoL trajectories differ between patients with and without diabetes and identify to what extent patient characteristics are related to poor QoL outcomes after total joint replacement (TKR). Multilevel modelling was used to analyse long-term QoL patterns of patients undergoing TKR between 2006 and 2011. Patient-reported QoL at baseline and up to 5 years post-surgery were included. Of the 1553 TKR patients, one-fifth (*n* = 319) had diabetes. Despite there being no significant differences in QoL at baseline, patients with diabetes consistently reported lower QoL (on average by 0.028, *p* < 0.001) and did not improve to the same level as patients without the disease following surgery. Compared to males, females had significantly lower QoL (by 0.03, *p* < 0.001). Other baseline patient characteristics associated with important differences in QoL included presence of respiratory disease and mental health disorder. Patients with diabetes exhibit significantly poorer QoL compared to patients without diabetes, particularly among females. Knowledge of risk factors that impact on QoL can be useful for clinicians in identifying characteristics related to poor QoL outcomes and be used to guide patient-centered care.

## 1. Introduction

The global prevalence of diabetes has almost tripled in the last two decades and is the highest among those over the age of 65 years [1]. Among those with diabetes, 50% also suffer from arthritis [2], for which many will require surgery for relief of symptoms. Total knee replacement (TKR) is now one of the most common surgical procedures [3] and the rate of surgeries performed each year continues to grow [4,5]. TKR is proven to be an effective intervention for severe osteoarthritis by improving patients’ pain, mobility, well-being and quality-of-life (QoL) [6,7,8].

Patient-reported outcome measures (PROMs) are important measures of clinical care as they provide valuable information on the effectiveness of the surgical intervention from the patient’s perspective. The most prominent use of PROM data is in estimating quality-adjusted life years for informing the value of an intervention, through economic evaluations such as cost-effective analyses. As practices shift towards patient-centred care and QoL, PROMs facilitate shared decision making with patients to tailor care based on individual needs. These measures can be used to track patient progress and the disease impact on patients’ overall QoL.

Impairment in health, functional capacity and pain are some of the mains reasons patients seek surgical care. PROMs in the form of generic QoL instruments, such as the Short Form 12 Health Survey (SF-12), are valuable tools to assess patients’ response to treatment. While significant improvement to patients’ QoL is commonly observed after TKR, patients with diabetes frequently report lower QoL than the general population [9,10,11,12]. Evidence regarding surgical complications and outcomes in relation to TKR in the presence of diabetes remains controversial [13,14,15,16,17,18]. Some studies have shown the risk of infections, revisions and surgical complications to be greater in patients with diabetes [13,14,15,16], while others have demonstrated otherwise, showing no significant differences in revision, surgical complication rates and functional outcomes of TKR between patients with and without diabetes [17,18].

The majority of these studies assess the quality of surgical care through traditional clinical outcome measures. It is unclear whether patient-reported QoL trajectories differ between patients with and without diabetes after TKR. The average summary scores reported in the literature provide limited information about individual change and are usually over a short period post-surgery. A better understanding of longer-term QoL trajectories can be useful in guiding diabetes care and can help patient and physician understand the impact of surgery on patient well-being [19]. Using annual QoL measures collected from a large registry cohort of TKR patients over a 5-year period, we examined if and to what extent QoL trajectories differ between patients with and without diabetes and what patient characteristics or subgroups were related to poor QoL outcomes.

## 2. Methods

### 2.1. Data Source and Study Population

The St. Vincent’s Melbourne Arthroplasty Outcomes (SMART) Registry is a repository of clinical and patient reported outcomes for all patients who undergo elective hip and knee replacement at the study institution. Prospectively collected baseline data on patients who underwent TKR between 1 January 2006 and 31 December 2011 were available and this included age, sex, body mass index (BMI), smoking status and American Society of Anesthesiologist (ASA) Physical Status Classification and self-reported co-morbidities including diabetes. Socioeconomic status was collected according to the Socio-Economic Index for Areas (SEIFA) [20] and geographical accessibility index (ARIA+) [21] reflecting rurality. Other clinical variables included contralateral knee surgery and radiographic osteoarthritis severity using the Kellgren–Lawrence grading system.

Patients were required to have baseline QoL and at least one follow-up post-surgery to be included in the analysis. Individuals were excluded if they underwent early revision or died within 2 years of surgery. For individuals that underwent staged bilateral knee surgery during the study period, only the most recent TKR was included in the analysis. All patients were followed-up for up to 5 years.

### 2.2. Quality-of-Life Measurements

Patients completed SF-12 surveys within 12 weeks prior to surgery and annually post-operatively. Baseline and annual QoL scores up to 5 years post-surgery were analysed. SF-12 responses were transformed into utility values between 0 and 1, where 0 is equivalent to being ‘dead’ and 1 is equivalent to ‘full health’, using the published Brazier algorithm [22]. The algorithm is widely used to score SF-12 responses in clinical trials, outcomes assessments and economic evaluations.

### 2.3. Diabetes Classification

Patients were classified as diabetes or no diabetes based on self-reported information collected at baseline prior to surgery. Patients identified to have diabetes were further verified through checks of their patient medical records for information on anti-diabetic medication use (none, oral or subcutaneous) and glycated haemoglobin A1c (HbA1c) collected within 6 months of the date of surgery. Patients with diabetes were then further classified as having adequate glycaemic control (HbA1c < 7.0% (53 mmol/mol)) and poor control (HBA1c ≥ 7.0% (53 mmol/mol)).

### 2.4. Statistical Analysis

Differences in proportions between patients with diabetes and no diabetes were compared using the Pearson’s chi-squared test and paired *t-*tests for continuously distributed variables. Multilevel modelling was used to determine whether changes in QoL differed depending on diabetes status. This approach was used in this study as it can account for the longitudinal nature of the data, assess patterns of change of repeated measures over time, both within and between patients, and account for missing values [23,24]. This modelling approach can produce more robust coefficients compared to standard cross-sectional techniques as it allows for a flexible method of modelling within-cluster correlation; i.e., account for the correlation between QoL measures of individuals over time [23]. Time was modelled as a categorical predictor to allow for the flexibility in capturing QoL patterns over time and to facilitate comparisons across time points [25].

Diabetes status was included in the model as a main effect, and an interaction term with time was included if interaction terms were significant. The analysis was conducted for both males and females separately and combined, with and without controlling for possible confounders including age at surgery, sex, BMI, smoking status, radiographic osteoarthritis severity, existing co-morbidities, rurality and socio-economic status. Variables included in the final model were those variables that demonstrated evidence of significant association with QoL utility values (*p* < 0.05) identified using backwards stepwise elimination and cross validated using forwards stepwise selection. Separate models were also fitted to assess if QoL trends differed between patients on different types of antidiabetic medications and by glycaemic control. All analyses were conducted using Stata SE14 (StataCorp, College Station, TX, USA), employing Stata command MIXED for multilevel mixed-effects linear regression.

## 3. Results

**Figure 1:**
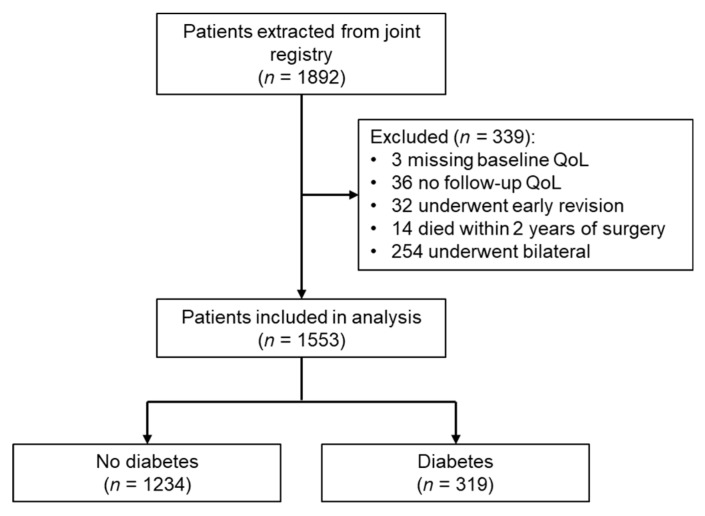
Flow diagram of patients included in the longitudinal analysis. QoL: quality-of-life.

A total of 1892 patients were identified from the registry. Patients were excluded if they had missing baseline utility score (*n* = 3), no follow-up utility scores (*n* = 36), underwent early revision (*n* = 32) or died within 2 years of surgery (*n* = 14). For individuals that underwent bilateral knee surgery during the study period (*n* = 254), only the most recent TKR was included in the analysis. After excluding 339 cases, 1553 TKR patients were included in the analysis (Figure 1). At five-year follow-up, 1218 (78.43%) patients had complete SF-12 responses at all six time points (including baseline).

Of the 1553 TKR patients, approximately one-fifth (*n* = 319) were identified to have diabetes. Table 1 summarizes the baseline characteristics of all patients according to diabetes status. Patients with diabetes were observed to be more likely to have higher BMI, report co-existing cardiovascular disease and scored higher on the ASA scale. Apart from these characteristics, there were no significant differences between other characteristics. Of note, both groups had similar mean baseline QoL utility values.

Among patient with diabetes, 203 patients (63.64%) and 31 (9.72%) were on oral and subcutaneous medications, respectively, while the remaining 85 (26.65%) were not on any medication. Information on HbA1c was available for 159 patients (49.84%). Among these patients, 99 (62.26%) had adequate glycaemic control (mean HbA1c was 6.34% (46 mmol/mol) (SD, 0.43)) and the remaining were classified as having poor control with mean HbA1c of 8.21% (66 mmol/mol) (SD, 1.21).

**Figure 2:**
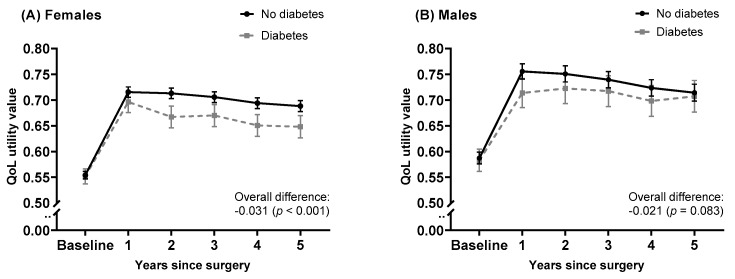
Long-term patterns of QoL utility value changes in total joint replacement (TKR) patients in (**A**) females and (**B**) males. The solid black lines represent no diabetes group; the grey dotted lines represent the diabetes group. Data points represent the time coefficients for each group predicted by the multilevel model adjusted for covariates. The error bars represent the 95% confidence intervals. QoL: quality-of-life.

Figure 2 shows the patterns of quality-of-life over 5 years from pre-surgery to 5-years post-surgery of patients by diabetes status and sex. In general, QoL improved markedly by 1-year post-surgery and plateaued in subsequent years. Despite both groups starting out with the same level of QoL at baseline, results from the multilevel model indicated that patients with diabetes consistently report lower QoL (on average by 0.028, *p* < 0.001) and did not improve to the same level as patients without the disease (Table 2). There were also evident differences between males and females (Figure 2). Females were found to have significantly lower QoL (by 0.030, *p* < 0.001) compared to males and the impact of diabetes on QoL was much more pronounced in females than in males. There were observable differences between the patterns of recovery between females and males. Females with and without diabetes have the same level of improvement up to 1 year post-surgery, however, their QoL trajectories diverge in subsequent years, resulting in a significant difference in QoL between those with and without diabetes. Contrarily, among males, those with diabetes achieve less improvement at 1-year post-surgery than those without diabetes but this difference reduces in subsequent years. Other risk factors associated with important differences in QoL included pre-existing respiratory or mental health conditions, ASA score, rurality and aetiology of disease (see Table 2).

**Figure A1:**
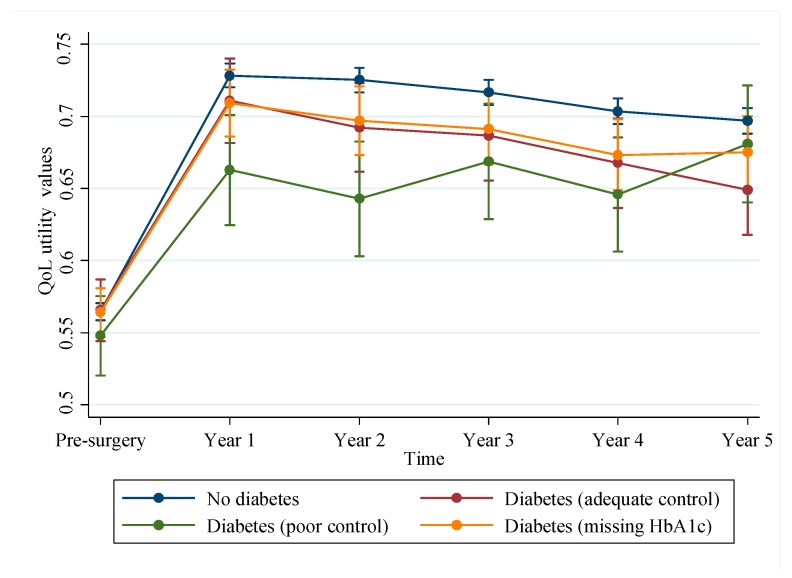
Patterns of QoL utility value changes in TKR patients across no diabetes and diabetes group. Those in the diabetes group were further sub-grouped into patients with poor (HbA1c ≥ 7%) and adequate glycaemic control (HbA1c < 7%), and those with missing HbA1c values. Data points represent the time coefficients for each group predicted by the multilevel model adjusted for covariates. The error bars represent the 95% confidence intervals. HbA1c: glycated haemoglobin, QoL: quality-of-life, TKR: total knee replacement.

**Figure A2:**
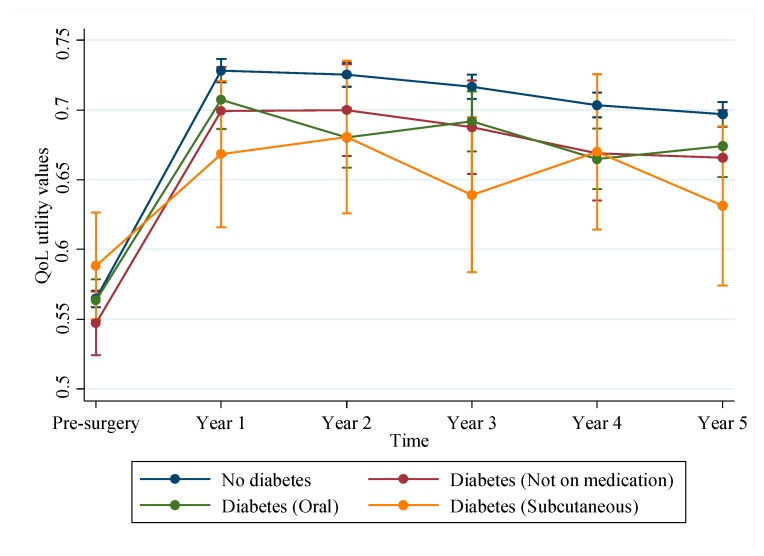
Patterns of QoL utility value changes in TKR patients across no diabetes and diabetes group. Those in the diabetes group was further categorised by the types of anti-diabetic medication use. The data points represent the time coefficients for each group predicted by the multilevel model adjusted for covariates. The error bars represent the 95% confidence intervals.

Subgrouping by glycaemic control (HbA1c) and medication types did not reveal any statistically significant differences in QoL trends among patients with diabetes (Figure A1 and Figure A2 in Appendix A).

## 4. Discussion

While studies examining QoL in patients with diabetes frequently report lower QoL than those without diabetes [10,11,12], there is much less literature reporting the long-term differences in QoL outcomes following a major surgical procedure such as TKR. In this longitudinal analysis of QoL outcomes after TKR, we found that despite there being no significant difference in QoL at surgery and achieving substantial improvement in QoL following TKR surgery, patients with diabetes do not achieve the same gains in health outcomes as patients without diabetes. This difference was most pronounced among females, with this patient subgroup persistently reporting lower QoL across the 5-year post-surgery period. These findings are useful in helping guide care among patients with diabetes and in facilitating discussions of expected outcomes and impact of surgery on their QoL. An important finding was the sex difference in outcomes highlighting the need to consider if females with diabetes should be managed differently in order to maximise their outcomes.

Studies examining functional outcomes after knee replacement found that patients with diabetes have lower ranges of motion and are at higher risk of limitations on daily activities and living post-surgery compared to patients without diabetes [16,26,27]. This may, in part, explain the poorer QoL observed in our study, and, if so, there may be a role for diabetes specific rehabilitation programs to maximise their outcomes. These could include lifestyle interventions to improve physical function [28] or exercise programs structured together with supervision to improve QoL [29]. Currently prescribed regimens tend not to discriminate between patient types, therefore, tailoring post-operative rehabilitation programs according to patients’ needs and relevant risk factors such as diabetes, other co-morbidities and by gender may be important. Because QoL utility values follow a rise and plateau pattern over time, the period after surgery (first year post-surgery) appears to be an important window to maximise patient outcomes from which the effects will plateau.

Although there are differences in patient-reported QoL across patient subgroups, it is also important to know if this translates into a meaningful difference. The minimal clinically important difference is commonly used to capture the smallest amount of change that would be considered beneficial to the patient [30]. The findings from this study indicate that improvements in QoL attained from TKR was substantial and significant, and that the differences observed between diabetes and no diabetes (on average 0.028, *p* < 0.001), and between female and male are important as they are within the range considered clinically important [31].

The sex differences reported in this study concur with existing literature which found females experiencing worse outcomes compared to males. This is not unique to knee surgery, as similar observations have been made in patients with stroke [32,33], rheumatoid arthritis [34] and in bipolar disorders [35]. The specific reasons for this are unclear but pain can have substantial impact on patient’s QoL outcomes and women with osteoarthritis may experience more pain and greater pain sensitivity which can translate into poorer QoL [36,37]. It may also be possible that women may be exposed to greater socioeconomic disadvantage than men which may have an impact on their recovery and QoL following surgery [38]. It would be important for future research to examine this to aid our understanding of differences in outcomes after TKR and to identify contributors to sex differences. Particular attention should also be paid to modifiable risk factors to poor response. Comorbidities in diabetes patients have been found to be associated with lower QoL and its negative impact on QoL increases with the number of comorbidities or a comorbidity index/score [39,40]. In this study, we found that patients reporting conditions that are treatable such as respiratory and mental health disorders are at significant risk of reporting poorer QoL (Table 2). This provides important information for clinicians to identify patients reporting these conditions as strategies to mitigate these factors may show outcome benefits.

Patients with diabetes are often ‘optimized’ pre-operatively, starting in primary care, which includes attaining good glycaemic control, sufficiently managing other diabetes-related co-morbidities and ensuring careful planning of care at all stages of the patient pathway [41,42]. Given that a patient’s baseline QoL is likely to be strongly correlated with their subsequent QoL over the follow-up period [43], there is scope to leverage the use of PROMs to optimize patients’ well-being pre-operatively to improve post-surgical outcomes. A recent randomised controlled study investigating the efficacy of a mental health enhancement program prior to joint surgery found the program an effective strategy in improving pain and physical function among those at risk of poor response to surgery [44]. This therefore suggests that optimizing other aspects of patient’s well-being beyond medication management and glycaemic control could also be an important consideration in ensuring better QoL outcomes post-surgery.

In general, TKR is widely regarded as a cost-effective procedure. However, studies have shown that patients with diabetes are associated with longer length of hospitalization and increased costs [45,46]. Given that QoL utility values are a key component in health economic analyses for assessing the value of the intervention, our findings indicate that patients with diabetes, particularly females and those with poor glycaemic control are less likely to achieve the same value compared to patients without diabetes. This aligns with a recent study identifying diabetes and females to be predictors of low-value care from the patients and payers’ perspectives, respectively [47]. Therefore, cost-effectiveness results based on population averages may not adequately reflect the true value of the intervention and more needs to be done to identify vulnerable populations that require better care and quantify the value of intervening. This can be important as healthcare systems are transitioning from volume- to value-based health care and emphasis has been placed on optimizing patient outcomes and experience [48]. The regression coefficients presented in this paper can be used to derive QoL utility values to assess the cost-effectiveness of specific subgroup populations.

Our study has several limitations. Patients included in this analysis were from a single institution which can limit the generalisability of the findings. However, the demographics of patients in this study closely reflect those reported in our National Joint Replacement Registry [5]. It was difficult to distinguish between type 1 and type 2 among diabetes patients based on the information captured in the registry; thus, it is unclear if QoL trajectories between these subgroups would be different, which warrants further research. A substantial amount of HbA1c information was missing as these were not documented in patients’ medical records which limited the interpretation of our results by HbA1c subgroups. While we do not know the reason for this missing information and it is uncertain if the missingness is related to an acknowledgement of good glycaemic control, this highlights the need for protocolised screening of diabetes and hyperglycaemia (with or without diagnosis of diabetes) as both are known risk factors for poor outcomes post-surgery [49,50].

## 5. Conclusions

Patients with diabetes exhibit significantly poorer QoL compared to patients without diabetes following TKR and this is emphasized in females. These findings highlight the need for a better understanding of patient and physiologic differences and for tailoring management to optimise patient outcomes. Knowledge of risk factors that impact on QoL after TKR may be used to guide patient-centered care.

## Figures and Tables

**Table 1:** Demographic and clinical characteristics according to diabetes status.

	No Diabetes		Diabetes		*p*-Value for Difference
	*n*	%	*n*	%	
Demographics	1234	79.46	319	20.54	
Age (SD)	69.90	8.73	70.67	7.74	0.149
Female	838	67.96	209	65.31	0.416
Smoking status					0.321
No	840	68.05	225	70.53	
Ex	303	24.57	78	24.45	
Yes	91	7.38	16	5.02	
SEIFA					0.400
1–5	453	36.71	109	34.17	
6–10	781	63.29	210	65.83	
Rurality					0.093
Metropolitan	1015	82.24	275	86.21	
Regional	219	17.76	44	13.79	
Clinical characteristics					
BMI					<0.001
<30	449	36.39	64	20.06	
30–35	406	32.9	104	32.6	
35–40	238	19.29	104	32.6	
40+	141	11.43	47	14.73	
Aetiology					0.067
Osteoarthritis	1148	93.03	308	96.55	
Other *	86	7.97	11	3.44	
Kellgren and Lawrence score †					0.677
≤3	597	48.54	159	49.84	
4	633	51.46	160	50.16	
Bilateral surgery	196	15.88	52	16.3	0.856
Reported co-morbid conditions					
Cancer	108	8.75	20	6.27	0.151
Cardiovascular	984	79.74	297	93.1	<0.001
Respiratory	225	18.23	57	17.87	0.88
Mental health disorder	223	18.07	71	22.26	0.089
Pre-operative status					
ASA					<0.001
1/2	773	62.64	128	40.13	
3/4	461	37.36	191	59.87	
Patient-reported QoL (SD)	0.57	0.11	0.56	0.11	0.138

* Other combines rheumatoid arthritis and avascular necrosis. † KL score missing for four patients. ASA: American Society of Anaesthesiologist (ASA) Physical Status Classification, BMI: body mass index, Ex: ex-smoker, QoL: quality-of-life, SEIFA: Socio-economic Index for Areas.

**Table 2:** Effect of patient characteristics on changes in QoL over time: coefficients estimated from multilevel regression model.

Description	All				Female				Male			
	Coef.	95%CI		*p*-Value	Coef.	95%CI		*p*-Value	Coef.	95%CI		*p*-Value
Year since surgery												
1	0.164	0.155	0.172	<0.001	0.161	0.151	0.172	<0.001	0.168	0.153	0.184	<0.001
2	0.161	0.152	0.170	<0.001	0.159	0.148	0.170	<0.001	0.164	0.147	0.180	<0.001
3	0.152	0.143	0.161	<0.001	0.152	0.141	0.162	<0.001	0.152	0.136	0.169	<0.001
4	0.139	0.129	0.148	<0.001	0.140	0.129	0.151	<0.001	0.136	0.119	0.153	<0.001
5	0.132	0.123	0.142	<0.001	0.134	0.123	0.146	<0.001	0.127	0.109	0.145	<0.001
Diabetes	−0.003	−0.017	0.010	0.655	−0.002	−0.018	0.014	0.792	−0.004	−0.029	0.020	0.719
Diabetes × year interaction												
1#Diabetes	−0.024	−0.043	−0.005	0.015	−0.017	−0.041	0.007	0.159	−0.037	−0.071	−0.003	0.031
2#Diabetes	−0.036	−0.056	−0.017	<0.001	−0.044	−0.068	−0.020	<0.001	−0.024	−0.059	0.011	0.186
3#Diabetes	−0.028	−0.048	−0.008	0.006	−0.033	−0.058	−0.009	0.007	−0.018	−0.053	0.018	0.321
4#Diabetes	−0.034	−0.055	−0.013	0.001	−0.041	−0.066	−0.016	0.001	−0.021	−0.057	0.016	0.262
5#Diabetes	−0.026	−0.047	−0.005	0.016	−0.038	−0.063	−0.013	0.003	−0.003	−0.041	0.035	0.892
Female	−0.030	−0.040	−0.020	<0.001	-	-	-	-	-	-	-	-
Respiratory	−0.020	−0.032	−0.008	0.001	−0.018	−0.032	−0.004	0.014	−0.026	−0.049	−0.002	0.031
Mental health disorder	−0.040	−0.052	−0.028	<0.001	−0.034	−0.048	−0.020	<0.001	−0.053	−0.076	−0.030	<0.001
ASA												
1/2	Ref				Ref				Ref			
3/4	−0.028	−0.037	−0.018	<0.001	−0.028	−0.039	−0.016	<0.001	−0.026	−0.044	−0.009	0.004
Rurality												
Metropolitan	Ref				Ref				Ref			
Rural	0.031	0.018	0.043	<0.001	0.030	0.014	0.046	<0.001	0.032	0.012	0.053	0.002
Aetiology												
Osteoarthritis	Ref				Ref				Ref			
Other*	−0.030	−0.049	−0.011	0.002	−0.020	−0.042	0.003	0.083	−0.055	−0.093	−0.017	0.005
Constant	0.601	0.578	0.623	<0.001	0.571	0.562	0.581	<0.001	0.607	0.592	0.621	<0.001
Random effect												
Residual standard deviation at each time point (SE)												
0	0.108	0.002	-	-	0.105	0.002	-	-	0.115	0.004	-	-
1	0.148	0.003	-	-	0.147	0.003	-	-	0.150	0.005	-	-
2	0.152	0.003	-	-	0.150	0.003	-	-	0.156	0.005	-	-
3	0.153	0.003	-	-	0.152	0.003	-	-	0.155	0.005	-	-
4	0.151	0.003	-	-	0.150	0.003	-	-	0.154	0.005	-	-
5	0.152	0.003	-	-	0.150	0.003	-	-	0.156	0.005	-	-

* Other combines rheumatoid arthritis and avascular necrosis. ASA: American Society of Anaesthesiologist (ASA) Physical Status Classification, CI: confidence interval, Coef: coefficient.
